# Concomitant Administration of Vancomycin with a High Dose of Meropenem May Cause Acute Kidney Injury

**DOI:** 10.1155/2024/7956014

**Published:** 2024-06-20

**Authors:** Yoshiro Sakai, Seiji Karakawa, Takato Koutaki, Kyoko Higuchi, Aya Hashimoto, Hiroshi Watanabe

**Affiliations:** ^1^Department of Pharmacy, Kurume University Hospital, Kurume, Japan; ^2^Department of Infection Control and Prevention, Kurume University School of Medicine, Kurume, Japan; ^3^Department of Neurosurgery, Kurume University School of Medicine, Kurume, Japan

## Abstract

Coadministering two different classes of antibiotics as empirical therapy can be critical in treating healthcare-associated infections in hospitals. Herein, we report a case of acute kidney injury (AKI) caused by coadministration of vancomycin with high-dose meropenem that manifested as a rapid increase in serum creatinine levels and an associated increase in vancomycin trough concentrations. The patient was diagnosed with meningioma at 50 years and was followed up regularly. The patient underwent surgery and antibiotic treatment between 63 and 66 years for suspected meningitis and pneumonia. Coadministration of vancomycin with high-dose meropenem (6.0 g/day) caused AKI; however, no AKI occurred when vancomycin was administered alone or with a low dose of meropenem (1.5 or 3.0 g/day). To our knowledge, this report is the first to show that administering different dosages of meropenem in combination with vancomycin may contribute to the risk of developing AKI. We suggest that coadministered vancomycin and high-dose meropenem (6.0 g/day) may increase the risk of AKI. Our report adds to the limited literature documenting the coadministration of vancomycin with varying doses of meropenem and its impact on the risk of AKI and highlights the importance of investigating AKI risk in response to varying dosages of meropenem when it is coadministered with vancomycin.

## 1. Introduction

Vancomycin, a glycopeptide antibiotic, is widely used in clinical practice to treat infections caused by Gram-positive bacteria resistant to many other antibiotics, including methicillin-resistant *Staphylococcus aureus* (MRSA) [1]. Meropenem, a broad-spectrum antibacterial agent of the carbapenem family, is used for the treatment of a broad range of serious infections [2]. The combination of vancomycin and meropenem is occasionally used as an empirical therapy for severe infections [3]. Similar to the combination of vancomycin and tazobactam/piperacillin, the concomitant use of vancomycin with meropenem has been reported to cause acute kidney injury (AKI), with the incidence rate of AKI varying from 3.6 to 27.7% [3–10]. For treating bacterial meningitis, meropenem is used at a high dose (2.0 g every 8 h or 6.0 g/day), compared to the dose used for other mild to moderate infections [3].

Herein, we report a case of AKI with a high trough concentration of vancomycin, resulting from the combination of vancomycin and high-dose meropenem, that is, 6.0 g/day. In this case, vancomycin was administered alone and in combination with various doses of meropenem (1.5 or 3.0 g/day) for different infections, including meningitis, from the ages of 63 to 66 years.

## 2. Case Presentation

A female patient was diagnosed with meningioma at the age of 50 years and was followed up with regular examinations. At 63 years (body weight: 64.0 kg), the patient experienced dizziness while walking and was referred to our hospital because of hearing impairment in the left ear and inability to walk. Bacterial meningitis was suspected after surgery and was treated with intravenous meropenem at a dose of 0.5 g every 8 h (1.5 g/day) over 1 h and was coadministered with intravenous vancomycin at a dose of 1.0 g every 12 h over 1 h. Additional concomitant medications administered at the start of treatment were valsartan (80 mg/day) and trichlormethiazide (1 mg/day). Concomitant treatment with two antibiotics was continued for 19 days. During treatment, the dose of meropenem remained unchanged, and that of vancomycin was adjusted based on the trough concentration. The blood trough concentration on day 15 was 16.6 *μ*g/mL. No AKI was observed with this treatment. Meningitis was ruled out, and the treatment was completed. Vancomycin was administered for 19 days.

At the age of 65 years (body weight: 58.3 kg), the patient reported dysphagia, and because tumor growth was observed, re-excision was performed. The surgery was performed endoscopically and openly. Postoperatively, the patient was suspected of having bacterial meningitis and was treated with meropenem over 1 h (2.0 g every 8 h or 6.0 g/day) and vancomycin over 1 h (1.0 g every 12 h). Olmesartan (20 mg/day) was the only concomitant drug administered at the start of treatment. Treatment was discontinued because the patient developed AKI 3 days after concomitant treatment with vancomycin and meropenem, and it manifested as a rapid increase in serum creatinine levels and an associated increase in vancomycin trough concentrations. The vancomycin trough concentration on day 3 of concomitant use was 55.2 *μ*g/mL. Bacterial meningitis was ruled out, and antibiotic therapy was halted. After stopping the antibiotics, the renal function of the patient returned to pretreatment levels.

At the age of 66 years (body weight: 53.0 kg), treatment with vancomycin (0.5 g every 12 h over 1 h) was started for MRSA pneumonia. Additional concomitant medications administered at the start of vancomycin treatment were amlodipine (10 mg/day), sennoside (24 mg/day), and esomeprazole (10 mg/day). The dose of vancomycin was increased during the treatment, and the final treatment included a dose of 0.9 g every 12 h over 1 h. The patient did not develop AKI during treatment with vancomycin alone, and the vancomycin trough concentration on day 11 was 10.1 *μ*g/mL. Vancomycin was administered for 12 days.

**Figure 1:**
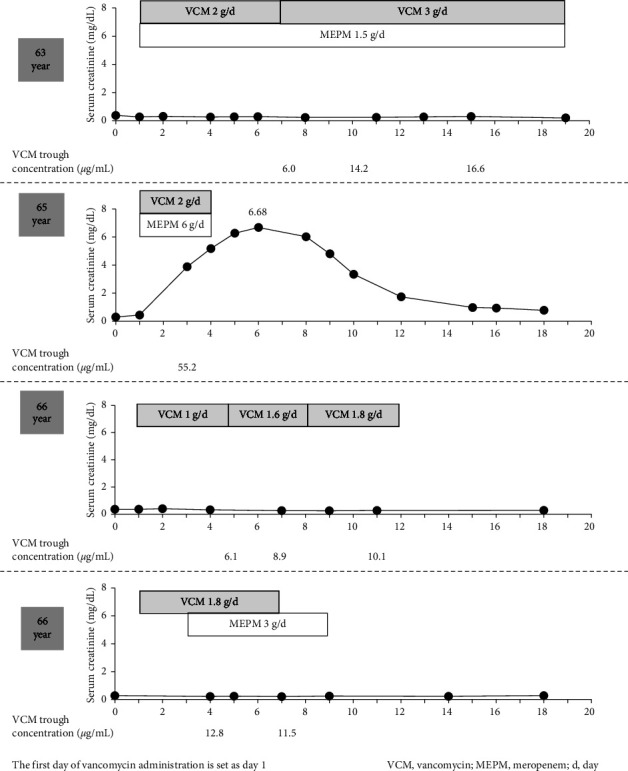
Changes in antimicrobial therapy, serum creatinine level, and vancomycin trough concentration.

In addition, at the age of 66 years (body weight: 51.5 kg), the patient underwent shunt surgery for hydrocephalus and started treatment with vancomycin (0.9 g every 12 h over 1 h) for MRSA colonization before surgery. Initial concomitant medications were amlodipine (10 mg/day), sennoside (24 mg/day), esomeprazole (10 mg/day), ursodeoxycholic acid (600 mg/day), and magnesium oxide (990 mg/day). Postoperatively, the patient was suspected of having a wound infection, and meropenem was initiated at a dose of 1.0 g every 8 h over 1 h or 3.0 g/day. No AKI occurred during the antibacterial drug treatment, and the trough concentration of vancomycin on day 7 was 11.5 *μ*g/mL. Vancomycin and meropenem were administered for 7 days.  Figure 1 shows the changes in renal function after four rounds of antibacterial drug treatment.

Vancomycin was measured by the latex agglutination turbidimetry method using blood samples from patients.

## 3. Discussion

To our knowledge, this is the first case report to show that different doses of meropenem in combination with vancomycin may affect the development of AKI. In this case, four vancomycin treatments were administered between 63 and 66 years. Among these four treatments, AKI appeared only once when meropenem was coadministered at 6.0 g/day. In the other three cases of vancomycin treatment, wherein AKI did not occur, vancomycin was administered alone or concomitantly with a meropenem dosage of 1.5 or 3.0 g/day. Therefore, we suggest that concomitant use of vancomycin and high-dose meropenem, that is, 6.0 g/day, may increase the risk of AKI.

The concomitant use of vancomycin with piperacillin/tazobactam has been reported to increase the risk of AKI compared with vancomycin treatment alone or concomitantly with other *β*-lactams [3–10], suggesting that its concomitant use needs to be minimized [11]. Moreover, if concomitant use of vancomycin with other antibiotics is necessary, cefepime, or antipseudomonal carbapenem should be used instead of piperacillin/tazobactam [12]. Concomitant administration of drugs other than antibacterial drugs may also contribute to the development of AKI. Angiotensin receptor blockers (ARBs) were concomitantly administered with vancomycin and meropenem at the time of AKI. Although the preoperative use of ARBs is associated with the risk of AKI [13], we believe that the coadministration of ARBs had little effect on the development of AKI in this case because the concomitant use of ARBs with vancomycin or with a meropenem dosage of 1.5 or 3.0 g/day did not cause AKI. In addition, surgery is considered to be a risk for AKI [14], but we consider that the surgery had little impact, as the operation was completed on time, there was not much bleeding and no intraoperative hypotension was observed.

There are no case reports of AKI caused by the coadministration of vancomycin with high-dose meropenem. For cases with AKI, where a combination of vancomycin and meropenem was used, the dose of meropenem remains unknown, and the risk of using different dosages of meropenem has not been reported [3–5, 7]. In this case, high-dose meropenem, that is, 6.0 g/day, emerged as a risk factor compared with the other doses of meropenem used in combination therapy (1.5 or 3.0 g/day) and vancomycin monotherapy. Cases of AKI with meropenem treatment alone have been reported along with other side effects; however, the dose was not mentioned [15–17]. In Japan, high-dose meropenem, including concomitant use with vancomycin, was used in treating four patients with adult bacterial meningitis without the development of AKI [18]. Regarding nephrotoxicity caused by meropenem, increased blood concentration is considered associated with AKI [19]. In this case, AKI occurred only when high-dose meropenem was administered. We believe that high-dose meropenem increased the blood concentration of meropenem and caused nephrotoxicity, and since vancomycin was administered concomitantly, an additive nephrotoxicity effect occurred, which was accompanied by an increase in the blood concentration of vancomycin. We did not consider that coadministered vancomycin and high-dose meropenem will necessarily cause AKI; however, we believe that it may be a risk factor for AKI. Therefore, we acknowledge that it is necessary to investigate the occurrence of AKI in response to various doses of meropenem wherein vancomycin and meropenem are used concomitantly in a large-scale study in the future.

## 4. Conclusions

In summary, we suggest that the risk of AKI needs to be monitored in cases were where vancomycin and meropenem are used concomitantly, and high-dose meropenem (6.0 g/day) may require careful monitoring. Our report adds to the limited literature documenting the coadministration of vancomycin with varying doses of meropenem and its impact on the risk of AKI. Moreover, it highlights the risk of AKI associated with the coadministration of vancomycin with a high dose of meropenem, highlighting the importance of investigating the risk of AKI in response to the varying dosages of meropenem when coadministered with vancomycin.

## Data Availability

The data used to support the findings of this study are available from the authors upon reasonable request.
